# AI Governance: A Challenge for Public Health

**DOI:** 10.2196/58358

**Published:** 2024-09-30

**Authors:** Jennifer K Wagner, Megan Doerr, Cason D Schmit

**Affiliations:** 1 School of Engineering Design and Innovation, Penn State University, University Park, PA, United States; 2 Department of Anthropology, Penn State University, University Park, PA, United States; 3 Institute for Computational and Data Sciences, Penn State University, University Park, PA, United States; 4 Department of Biomedical Engineering, Penn State University, University Park, PA, United States; 5 Rock Ethics Institute, Penn State University, University Park, PA, United States; 6 Penn State Law, Penn State University, University Park, PA, United States; 7 Huck Institutes for the Life Sciences, Penn State University, University Park, PA, United States; 8 Sage Bionetworks, Seattle, WA, United States; 9 Population Informatics Lab, Department of Health Policy and Management, School of Public Health, Texas A&M University, College Station, TX, United States

**Keywords:** artificial intelligence, legislation and jurisprudence, harm reduction, social determinants of health, one health, AI, invisible algorithms, modern life, public health, engagement, AI governance, traditional regulation, soft law

## Abstract

The rapid evolution of artificial intelligence (AI) is structuralizing social, political, and economic determinants of health into the invisible algorithms that shape all facets of modern life. Nevertheless, AI holds immense potential as a public health tool, enabling beneficial objectives such as precision public health and medicine. Developing an AI governance framework that can maximize the benefits and minimize the risks of AI is a significant challenge. The benefits of public health engagement in AI governance could be extensive. Here, we describe how several public health concepts can enhance AI governance. Specifically, we explain how (1) harm reduction can provide a framework for navigating the governance debate between traditional regulation and “soft law” approaches; (2) a public health understanding of social determinants of health is crucial to optimally weigh the potential risks and benefits of AI; (3) public health ethics provides a toolset for guiding governance decisions where individual interests intersect with collective interests; and (4) a One Health approach can improve AI governance effectiveness while advancing public health outcomes. Public health theories, perspectives, and innovations could substantially enrich and improve AI governance, creating a more equitable and socially beneficial path for AI development.

## Introduction

Artificial intelligence (AI)—engineered or machine-based systems that can generate outputs like predictions, recommendations, or decisions for a given set of objectives with varying levels of autonomy [1]–has potentially unlimited applications. For instance, AI can be used to identify social media content that is likely to increase user engagement, but this may inadvertantly contribute to unprecedented mental health challenges and despair, particularly among young girls [2-4]. AI can also identify consumers that are likely to successfully repay bank loans, potentially exacerbating disparities in wealth and homeownership [5]. Further, AI can easily generate new sharable content, including written, artistic, or video media (including deepfake videos designed to deceive voters in order to advance political objectives) [6]. These examples underscore how AI can significantly impact the social, economic, or political determinants of health—in other words, AI acts as a structural determinant of health [7].

AI, unfortunately, often serves as an unwitting obfuscation engine that structuralizes social, economic, and political determinants of health into the invisible algorithms that shape all facets of modern life [8]. Often, it is not clear why AI models make the decisions they do [9]. AI can ingest and categorize data much more quickly than humans, generating hypotheses directly from data. Further, AI’s use of data in secondary, noncontextual, and unpredictable ways, including the cryptic (and not so cryptic) biases in the data it ingests, can easily obscure the disparities it reifies, cementing health inequity in care pathways and public policy [10].

This said, AI has the potential to be a potent public health tool [11]. AI could tremendously benefit public health applications, from supporting precision medicine and precision public health to enhancing public health surveillance efficacy [11]. Moreover, just as AI could exacerbate existing inequities, it is equally true that AI systems can be engineered to maximize equity [12].

AI has the potential to serve as a structural determinant of health and help achieve health equity. Consequently, public health experts have an important perspective to contribute to the future governance of AI. In this article, we define “governance” as the collection of frameworks, laws, policies, and practices that guide the development and implementation of AI to ensure its responsible and ethical use. Many public health policy innovations have enormous potential to enhance the broader AI governance discussion. Below, we explore how insights from public health policy can be leveraged to create a more perfect AI governance framework that is better equipped to support population well-being and equity.

## Harm Reduction Can Inform Ongoing Governance Debates

AI governance strategy could benefit from a harm reduction approach. Harm reduction rests on the axiom that pragmatic solutions that reduce harm or produce new benefits might be preferable to an ideal solution that faces practical impediments. the ideal approach advocated by many—traditional regulation—might not be the most pragmatic. Thus, the harm reduction approach could be useful to frame the current global debate on AI governance strategies.

Broadly, an ideal AI governance approach is one that maximizes benefits and minimizes harms. However, the pacing problem—where technological development outpaces legislative responses—poses a significant regulatory challenge. For instance, regulatory penalties need to be tied to clearly defined conduct, and AI is notoriously difficult to define legally in part because the technology is evolving at a significant rate [13-16]. Legal definitions that are specifically tailored to current or anticipated AI systems and applications can provide AI users and developers clarity, but the pacing problem threatens to make such definitions quickly obsolete. Alternatively, broad (eg, technology-neutral) definitions provide for a flexible and inclusive governance scope, but they can be vague and create uncertainty for AI users and developers. Not surprisingly, such definitional issues were debated extensively during the negotiations of the EU AI Act [13-16]. Second, it is challenging to effectively balance the benefits and risks of AI when new benefits and risks are coming to light daily. Due to these challenges, there might not be a *perfect* approach that can be practically implemented.

The AI governance debate currently centers on two different approaches to balancing the benefit/harm equation. The first approach involves the use of traditional laws (eg, statutes and regulations) for AI governance. The EU AI Act is one of the first examples of a traditional regulatory approach to AI [17]. Traditional laws have enforcement mechanisms that can encourage compliance with regulatory standards. However, traditional laws are notoriously slow to adapt to rapidly evolving technologies (ie, the pacing problem). In contrast to traditional laws, “soft laws” can be adapted relatively quickly to rapidly changing technological contexts: a good match for the field of AI [18]. Soft laws are (often voluntary) standards and rules that are designed to guide practices within an industry or sector. However, soft laws often lack robust enforcement mechanisms that are available with traditional laws [19]. Despite this, soft law approaches have demonstrated remarkable effectiveness in analogous industries [20,21]. In the absence of widespread traditional regulation, over 600 “soft law” frameworks have been introduced to guide AI development and use, with a majority being international (39%), US (14%), and European (11%) frameworks [22]. Since each approach presents unique risks and benefits, harm reduction can be instructive to identify a pragmatic path forward.

Viewing these two alternatives with a harm reduction lens, a soft law approach likely has a greater upside as an initial AI governance strategy. The pacing problem presents an inherent and unavoidable challenge to a traditional regulatory approach to AI governance. Statutes or regulations that are enacted today could quickly become outdated, restricting beneficial AI applications or permitting harmful ones. The enforcement strengths of traditional laws could be meaningless if harmful AI develops within unknown legal loopholes. In contrast, soft laws’ primary strength–an ability to quickly adapt to changing contexts–is critically important to address the pacing problem. Soft law standards can adapt with technology to limit the most significant risks while still allowing beneficial technological developments [1]. While enforcement is a weak dimension of soft law approaches, several strategies can minimize this limitation.

For example, soft law standards for ethical AI could be incorporated into traditional regulatory frameworks to enable a collaborative governance scheme that would be rapidly adaptable to the changing AI environment while supporting hard law incentives and consequences for non-compliance [23]. Collaborative governance can occur where governments incorporate soft law standards and guidance into their hard law regulatory framework. A government regulator could deem a business compliant with a set of regulations, grant enforcement leniency, or exempt the business from certain regulatory standards if the business complies with soft law standards. For instance, US federal and state regulators use the “soft law” accreditation standards from the Joint Commission to facilitate the regulation of health care facilities [24]. Similarly, in our 2023 *Science* article, we proposed using licensing to (1) embed soft law within AI terms of use and (2) systematically pool individual enforcement rights in a quasi-governmental AI regulator [23]. In each of these cases, soft laws are essential to address harms that will be derived from the pacing problem.

An ideal AI regulatory approach would likely need (1) clear regulatory definitions, standards, and rules; (2) capacity to adapt to evolving AI risks and opportunities; and (3) robust enforcement mechanisms. The real-world challenges facing AI governance pose a daunting obstacle to traditional regulatory approaches. A harm reduction approach to AI governance may include initial prioritization of rapidly evolving soft law standards to guide AI development. Such an approach could be further enhanced with collaborative governance, tying soft law standards with “hard” law incentives, or other legal innovations to promote the use of responsible AI.

## Weighing Risks Through a Social Determinants of Health Lens

Axiomatically, a desirable AI governance framework is one that attempts to maximize benefits while minimizing risks, yet some benefits and risks are more difficult to weigh than others. For instance, racial bias is an unquestioned normative concern, but how does one weigh a risk of racial bias against a tangible benefit (eg, efficient deployment of health care staff)? In the absence of additional information, weighing such tradeoffs is a difficult task.

However, there is enormous potential for public health understanding of social determinants of health to better inform policymakers and AI users as they weigh benefits and risks. For instance, racial discrimination is recognized as a powerful social determinant of health with significant and measurable consequences. Public health scholarship can help policymakers and AI users understand the broad extent of the social, economic, and health harms of racial bias. This understanding is crucial for accurately balancing the anticipated risks of an AI application against its potential benefits. Similarly, public health scholarship understands inequity not merely as a normative problem, but as a significant social determinant of health. Understanding the social, economic, and health harms associated with inequity can be particularly useful for organizations as they consider different strategies to mitigate bias in AI models.

Consider a hypothetical situation with two different approaches for mitigating bias in an AI model. While it might be tempting to choose the approach with the greatest absolute impact on bias reduction, the best approach might be more nuanced. For example, an approach that does slightly worse at mitigating bias from an AI model might be superior if the remaining bias serves to increase the equity of the AI model overall. Understanding the harms associated with inequity could be crucial for conducting a utilitarian ethical analysis of the two bias mitigation strategies.

In summary, incorporating an understanding of the social determinants of health into the careful weighing of competing interests, risks, and benefits of AI applications is essential if policymakers are to formulate rules, guidelines, and standards that effectively minimize AI risks while maximizing its benefits.

## Public Health Ethics: A Toolset for AI Ethics

AI presents vexatious ethical issues. Many issues wrestle with apparent friction between individual interests and AI uses that could provide widespread public benefits. These are difficult questions, but again, public health insights provide new options to wade through these quagmires.

Public health organizations have long wrestled with ethical challenges where individual interests sometimes conflict with activities that would promote broad public benefits. In 1991, the Council of International Organizations of Medical Sciences acknowledged that traditional bioethics was an inadequate tool to resolve ethical challenges for studies involving “groups” of people [25,26]. Subsequently—and over several decades—public health ethics emerged as a field designed to wrestle with these challenges. The World Health Organization’s 2017 guidance on public health surveillance balanced ethical principles of common good, equity, respect for persons, and good governance to provide a path through many ethical challenges where individual privacy interests conflict with socially beneficial data uses [27].

Likewise, the AI governance debate faces similar challenges. For instance, AI raises significant concerns about individual privacy, while simultaneously demonstrating the potential for extensive social benefits. Policymakers have a significant challenge trying to find an appropriate balance [28-30]. In the 2022 Blueprint for an AI Bill of Rights, US President Joe Biden’s administration acknowledges that both individuals and communities have an interest in AI development, but it fails to provide a framework for navigating issues when those interests are competing [31]. These debates are still dominated by a bioethical paradigm that was developed to address abuses involving more personal relationships and activities (eg, doctor-patient and researcher-participant). While big data and AI increasingly implicate population scale activities and their impact, US policymakers have continued to go all-in on protective mechanisms derived from the bioethical paradigm (eg, notice and individual consent requirements), which privacy scholars find “mystifying” [32,33].

Former executive director of the Presidential Commission for the Study of Bioethical Issues, Lisa Lee has argued that public health ethics is crucial to fill an existing conceptual gap in ethical thinking between individual-focused biomedical ethics and environmental ethics [34]. For its part, AI—which does not fit neatly in either category—is in desperate need of a gap-filler. Public health ethics, with its broad focus that overlaps with individual, community, and environmental concerns, can be that gap-filler framework [35]. Taking a broad view of health (ie, “a complete state of physical, mental and social well-being”), public health ethics is a suitable tool to address many ethical AI issues that do not fit neatly within traditional biomedical or environmental ethics frameworks [34-36].

Currently, many AI governance debates are toiling through these difficult challenges. There is no need to recreate the wheel. Public health ethics may help provide a pathway, toolset, and language for AI developers to problem-solve and communicate ethical solutions, enabling purposeful, responsible, and conscientious AI to promote socially beneficial outcomes [27].

## One Health to Shape AI and AI to Shape One Health

One Health represents the idea that humans are intimately connected to—and their health is intrinsically intertwined with—that of non-human animals, plants, and the environment [37]. Deliberately approaching AI through a One Health lens could enable a meaningful recognition of an international human right to health by enabling integrated, transformative policy interventions to impel responsible AI across diverse sectors of society (and diverse data ecosystems) and better address complex health threats. If AI governance is adopted without adequately accounting for relational dynamics and concurrent needs (such as necessary data integration and coordination) to advance healthy humans, non-human animals, and environments, the governance could thwart existing One Health efforts or perhaps shorten the functional utility of the AI governance policy itself when these unintended consequences are apparent. For example, sector-specific AI rules could frustrate One Health approaches for combating antimicrobial resistance or infectious disease surveillance/prevention that require collaboration and coordination across medical, agricultural, veterinary or husbandry, and other industries. Moreover, some AI activity or use that is deemed low risk in one context could be high risk when another context is considered. Existing disparities and inequities (of varying scales) can be perpetuated (if not reinforced) in the absence of a One Health approach to AI that aims not only to elevate solidarity and equity but also devise solutions that are, for example, fair to those regardless of geographic, economic, or other circumstance.

## AI Governance Is a Problem for Public Health, Not a Problem About AI

As an emerging structural determinant of health, AI presents both challenge and opportunity. Simply put, regulation of AI without public health would be disastrous for public health. Certainly, there are those that have framed AI as posing an existential threat, but short of those apocalyptic scenarios there could be monumental insidious effects on population health, including widening inequities and disparities. Moreover, public health representation in AI governance is necessary to ensure that beneficial AI tools are not inadvertently prohibited from public health applications [38].

Yet, public health representation in AI governance can support more effective, responsive, and equitable AI rules. Public health has a long history of developing policy innovations to address wicked societal problems. In this way, AI governance is a problem for public health leaders and scholars to help shape a better future for communities around the globe.

Despite the potential benefits of these public health perspectives, few AI soft law frameworks include public health voices [18,22,38]. For example, Gutierrez and Marchant [22] conducted a comprehensive review of the exploding landscape of soft law AI frameworks. Across the 638 frameworks reviewed, they identified 78 themes, only 5 of which were health related, and none of which focused on public health [22]. Nevertheless, there are some promising signs that public health perspectives might be increasing in AI governance debates [39]. For example, the United Nations recently convened an advisory body on AI that comprises a few members with public health experience [40], the impact of which is already apparent with explicit nods to public health’s importance (eg, noting AI’s potential to “transform public health,” and the need to regularly assess the structural and potential “critical social impacts” of AI on public health) [41]. Despite these examples of recognition of the possible impact of AI on public health efforts, we are not aware of any deliberate application of a public health lens when developing AI governance. More public health voices are likely needed to integrate existing public health knowledge and tools in the global efforts to create AI standards that can support socially beneficial outcomes and general wellness.

## Conclusion

AI governance presents a monumental and pressing challenge to governments around the globe as as competitive forces incentivize reckless AI development over a more responsible, purposeful approach. It is increasingly likely that policy innovations will be needed to address the unique challenges AI poses. Innovations in public health policy and relevant tools can help policymakers navigate the complex and challenging AI governance issues and could be critical in developing a framework that is most effective in maximizing the benefits of AI while minimizing its risks. Yet, many existing AI governance efforts lack public health perspectives. Regulation of AI without public health would be disastrous for public health. However, regulating AI with public health is the key to unlocking AI’s promised societal benefits.

## Abbreviations

AI: artificial intelligence
